# 

**Published:** 1862-01

**Authors:** 


### MEDICAL CRITIC

AND

### PSYCHOLOGICAL

### JOURNAL.

EDITED BY

FORBES WINSLOW, M.D., D.C.L. Oxon.

VOL. II.

Surgeon Genl's Office. LIBRARY. 4108 Washington, D.C.

LONDON:
JOHN W. DAVIES, 54, PRINCES STREET,
LEICESTER SQUARE.

EDINBURGH: MACLACHAN AND CO. DUBLIN: FANNIN AND CO.

M DCCC LXII.

WI
ME 286

LONDON:
SAVILL AND EDWARDS, PRINTERS, CHANDOS-STREET,
COVENT GARDEN.

### WORKS PUBLISHED DURING THE QUARTER.

### 1. *Medicine.*

BARLOW.—A Manual of the Practice of Medicine. By G. H. Barlow, M.D. Second Edition, considerably enlarged. Fcap. 8vo, cloth, 12s. 6d.

BENNETT.—A Practical Treatise on Inflammation and other Diseases of the Uterus. By J. Henry Bennett, M.D. Fourth Edition, with numerous Additions. 8vo, cloth, 16s.

BUCKNILL and TUKE.—A Manual of Psychological Medicine; containing the History, Nosology, Description, Statistics, Diagnosis, Pathology, and Treatment of Insanity. By J. C. Bucknill, M.D., and D. H. Tuke, M.D. Second Edition, revised and enlarged. 8vo, cloth, 15s.

CLAY.—General and Medical Education; the Introductory Lecture delivered in Queen's College, Birmingham, on the Opening of the Winter Session, 1861-62. By John Clay. 8vo, 1s.

DOBELL.—Lectures on the Germs and Vestiges of Disease, and on the Prevention of the Invasion and Fatality of Disease by Periodical Examinations. By Horace Dobell, M.D. 8vo, cloth, 6s. 6d.

FLEISCHMANN.—Practical Medical Precepts for Plain People. A large sheet, 32 inches by 22 inches, 4d.

Guy's Hospital Reports. Edited by S. Wilks, M.D., and A. Poland, Esq. Third Series, Vol. VII., with Plates, Plain and Coloured, 7s. 6d.

JACKSON.—Medical Climatology: or, a Topographical and Meteorological Description of the Localities resorted to in Winter and Summer by Invalids of various classes, both at Home and Abroad. By R. E. Scoresby Jackson, M.D., F.R.S.E. With an Isothermal Chart. Post 8vo, cloth, 12s.

LEARED.—On the Sounds caused by the Circulation of the Blood. By Arthur Leared, B.A., M.D. Dub., M.R.C.P. Lond. 8vo, 1s.

LEE.—Bradshaw's Invalid's Companion to the Continent. By Edwin Lee, M.D. Second Edition, post 8vo, cloth, 10s.

MARTIN.—Influence of Tropical Climates in Producing the Acute Endemic Diseases of Europeans. Including Practical Observations on their Chronic Sequelæ under the Influences of the Climate of Europe. By Sir Ranald Martin, K.C.B., F.R.S. Second Edition, much enlarged, 8vo, cloth, 20s.

Medico-Chirurgical Transactions, published by the Royal Medical and Chirurgical Society of London. Vol. XLIV.; Second Series, Vol. XXVI. 8vo, cloth, 12s.

MOORE.—A Manual of the Diseases of India. By William James Moore, M.D. Fcap. 8vo, cloth, 5s.

MUNK.—The Roll of the Royal College of Physicians, London. Compiled from the Annals of the College, and from other Authentic Sources. By William Munk, M.D., Fellow of the College, &c. Vol. II. 8vo, cloth, 12s.

REYNOLDS.—Epilepsy: its Symptoms, Treatment, and Relation to other Chronic Convulsive Diseases. By J. Russell Reynolds, M.D. Lond., F.R.C.P. 8vo, cloth, 10s.

RICHARDSON.—The Asclepiad. A Serial. By Benjamin W. Richardson, M.A., M.D. Vol. I.—Clinical Essays. 8vo, cloth, 6s. 6d.

STRUTHERS.—Royal Colleges of Physicians and Surgeons under the New Medical Act. By John Struthers, M.D. 8vo, 1s.

UNDERWOOD.—Medical Student's Guide to the Translation of Prescriptions. By W. J. Underwood. New Edition, 18mo, 5s. 6d.

WEBB.—The Study of Medicine: its Dignity and Rewards. Being an Inaugural Address, delivered at the commencement of the Winter Session 1861-2, at the Grosvenor-place School of Medicine. By Francis C. Webb, M.D., M.R.C.P. 8vo, 1s.

### 2. *Surgery.*

ANDERSON.—The Parasitic Affections of the Skin. By T. M'Call Anderson, M.D., F.F.P.S. With Engravings on Wood. 8vo, cloth, 5s.

COOPER.—Cooper's Dictionary of Practical Surgery and Encyclopædia of Surgical Science. New Edition, by Samuel A. Lane, assisted by various eminent Surgeons. Vol. I., 8vo, cloth, 25s.

HULKE.—A Practical Treatise on the Use of the Ophthalmoscope. Jacksonian Prize Essay. By J. W. Hulke, F.R.C.S. Coloured Plates, 8vo, cloth, 8s.

SYME.—Observations on Clinical Surgery. By James Syme, F.R.C.S. Edin., &c. 8vo, cloth, 8s. 6d.

THOMPSON.—The Diseases of the Prostate, their Pathology and Treatment; comprising the Second Edition of "The Enlarged Prostate," and the Jacksonian Prize Essay of the Royal College of Surgeons. By Henry Thompson, F.R.C.S. With Plates, 8vo, cloth, 10s.

TUSON.—Spinal Debility, its Prevention, Pathology, and Cure, in Relation to Curvatures, Paralysis, Epilepsy, and various Deformities. By Edward W. Tuson, F.R.C.S. 8vo, cloth, price 5s.

YEARSLEY.—The Artificial Tympanum; a New Mode of Treating Deafness, when attended by Partial or Entire Loss of the Membrana Tympani, associated or not with Discharge from the Ear. By James Yearsley, L.R.C.P.E. Second Edition, price 1s.

### *Anatomy.*

BEALE.—On the Structure of the Simple Tissues of the Human Body, including the Connective Tissues. A Course of Lectures delivered at the Royal College of Physicians in April and May, 1861. With 10 Plates, and a Descriptive List of the Microscopical Specimens. By Lionel S. Beale, M.B., F.R.S., F.R.C.P. 8vo, cloth, 7s. 6d.

HOLDEN.—A Manual of the Dissection of the Human Body. By Luther Holden, F.R.C.S. Second Edition, with Engravings, 8vo, cloth, 16s.

JONES.—General Outline of the Organization of the Animal Kingdom; and Manual of Comparative Anatomy. By Rymer Jones. Third Edition, 8vo, cloth, 1*l.* 11s. 6d.

### 3. *Chemistry.*

GALLOWAY.—Chemical Tables. On Five Large Sheets, for Schools and Lecture Rooms. By Robert Galloway, F.C.S. Second Edition, 4s. 6d.

TAYLOR.—Human Manure, its Collection and Conversion to Guano. By Francis Taylor, M.R.C.S. 8vo, 1s.

